# Messages for patients and relatives from the 2021 update of the guideline on acute therapy and management of anaphylaxis

**DOI:** 10.1007/s40629-021-00185-3

**Published:** 2021-09-27

**Authors:** Johannes Ring, Kirsten Beyer, Tilo Biedermann, Andreas Bircher, Matthias Fischer, Thomas Fuchs, Axel Heller, Florian Hoffmann, Isidor Huttegger, Thilo Jakob, Ludger Klimek, Matthias V. Kopp, Claudia Kugler, Lars Lange, Oliver Pfaar, Ernst Rietschel, Franziska Rueff, Sabine Schnadt, Roland Seifert, Britta Stöcker, Regina Treudler, Christian Vogelberg, Thomas Werfel, Margitta Worm, Helmut Sitter, Knut Brockow

**Affiliations:** 1 Department Dermatology and Allergology Biederstein, Technical University Munich, Biedersteiner Straße 29, 80802 Munich, Germany; 2 Department of Pediatrics, Division of Pulmonology, Immunology and Critical Care Medicine, Charité—University Hospital Berlin, Berlin, Germany; 3 Department of Dermatology, University Hospital of Basel, Basel, Switzerland; 4 Clinic for Anaesthesiology, Intensive Care Medicine, Emergency Medicine and Pain Therapy, ALB FILS Hospitals Göppingen, Göppingen, Germany; 5 Department of Dermatology, University Hospital Göttingen, Göttingen, Germany; 6 Department of Anesthesiology and Operative Intensive Care Medicine, Medical Faculty, University of Augsburg, Augsburg, Germany; 7 Dr. von Hauner Children’s Hospital, Ludwig Maximilians University, Munich, Germany; 8 Department of Pediatrics, University Hospital Salzburg, Salzburg, Austria; 9 Department of Dermatology and Allergology, University Medical Center Gießen (UKGM), Justus-Liebig-University Gießen, Gießen, Germany; 10 Center of Rhinology and Allergology, Wiesbaden, Germany; 11 Pediatric Respiratory Medicine, Department of Pediatrics, Inselspital, Bern University Hospital, University of Bern, Bern, Switzerland; 12 St. Marien-Hospital Bonn, Bonn, Germany; 13 Section of Rhinology and Allergy, Department of Otorhinolaryngology, Head and Neck Surgery, University Hospital Marburg, Philipps-University Marburg, Marburg, Germany; 14 Department of Pediatrics, University Hospital Cologne, Cologne, Germany; 15 Department of Dermatology and Allergology, Hospital of the Ludwig Maximilians University, Munich, Germany; 16 German Allergy and Asthma Association, Mönchengladbach, Germany; 17 Institute of Pharmacology, Hannover Medical School, Hannover, Germany; 18 Medical practice for pediatrics and youth medicine, Poppelsdorfer Allee, Bonn, Germany; 19 Department of Dermatology, Venereology, and Allergology, Leipzig Interdisciplinary Allergy Center, University Hospital Leipzig, Leipzig, Germany; 20 Department of Pediatric Pneumology and Allergology, University Hospital Carl Gustav Carus, Technical University of Dresden, Dresden, Germany; 21 Immunodermatology and Experimental Allergology Unit, Department of Dermatology, Allergology, and Venereology, Medical University Hannover, Hannover, Germany; 22 Department of Dermatology, Venereology, and Allergology, Charité—University Hospital Berlin, Berlin, Germany; 23 Institute for Surgical Research, Philipps-University Marburg, Marburg, Germany

**Keywords:** Anaphylaxis, Insect venom, Drug, Food, Epinephrine auto-injector, Anaphylaxis passport/emergency plan, AGATE, COVID-19

## The most important aspects in a nutshell

- Anaphylaxis is always an emergency, so quick action is indicated.
- The medicines for immediate help must always be carried along.
- Get an anaphylaxis passport/anaphylaxis emergency plan.
- Recognize anaphylaxis: Familiarize yourself with the onset of symptoms and how anaphylactic reactions can manifest themselves (see Anaphylaxis Passport/Emergency Plan).
- Epinephrine is the emergency medication of choice: if in doubt, use the epinephrine auto-injector.
- If the epinephrine auto-injector has been used, always call 112.
- Practice handling the epinephrine auto-injector with the training pen.
- Inform your personal and social environment about anaphylaxis: Close relatives and caregivers should also be familiar with the use of emergency medications.
- You do not need to hide your condition: Be open about your allergy and the risk of anaphylaxis. Ask about relevant allergens when you visit a restaurant or go out for dinner.
- You are not alone; there are more and more people with anaphylaxis.
- Get support and more information through AGATE anaphylaxis training (www.anaphylaxieschulung.de) and the German Allergy and Asthma Association (www.daab.de).

## Development of the guideline

After the German Society of Allergology and Clinical Immunology (DGAKI) of 2017 had commissioned the working group “Anaphylaxis” to update the existing guideline on the acute treatment of anaphylaxis, the first task was to recruit experts from sibling societies as well as from neighboring disciplines, but also from neighboring countries and from patient organizations. It was possible to involve not only the subspecialty of allergology but also the (sub)specialities anesthesiology and intensive care medicine, dermatology, pediatrics, internal medicine, oto-rhino-laryngology, emergency medicine, pharmacology, pneumology, and theoretical surgery (for the methodology of guideline development). The allergological societies from Austria and Switzerland are represented as well as the patient organization Deutscher Allergie- und Asthmabund (DAAB). In continuous e‑mail exchange, but also in almost twice-yearly physical meetings in the context of conferences at various locations in Germany, the consensus was reached, which has been reflected in the now adopted guideline. Finally, a paragraph on the problem of allergy and COVID-19 was added. In January 2021, the guideline was made available in German and English to the two journals *Allergo Journal/Allergo Journal International* and *Allergologie/Allergologie select* and submitted to the Arbeitsgemeinschaft Wissenschaftlicher Medizinischer Fachgesellschaften (AWMF).

## Who is the guideline aimed at?

The guideline—as an abridged version of the medical guideline “Acute Therapy and Management of Anaphylaxis—Update 2021”—is primarily aimed at patients at risk of anaphylaxis as well as their relatives and persons who care for and look after affected persons at risk of anaphylaxis.

## What does “anaphylaxis” mean?

Anaphylaxis is the most severe form of an acute allergic immediate reaction that can be potentially life-threatening.

It manifests itself in various organ systems, most commonly the skin, respiratory tract, gastrointestinal tract and cardiovascular system. The symptoms usually start acutely and can progress rapidly. An anaphylactic reaction is classified as an emergency and must be treated immediately.

Depending on the organs affected and the intensity of the reaction, the treatment procedure is carried out by the patient himself, by caregivers or by medical personnel called in.

## How does an anaphylactic reaction manifest itself? What are the symptoms?

A distinction is made between objective symptoms, i.e., symptoms that can be seen or measured, such as hives, vomiting, or a drop in blood pressure, and subjective symptoms, i.e., symptoms perceived exclusively by the patient, such as itching on the palms of the hands and soles of the feet or in the genital area, metallic taste, itching or burning in the mouth area, or nausea. Often, at the onset of an anaphylactic reaction, these subjective general symptoms may manifest as milder discomfort and may be accompanied by feelings of anxiety or disorientation. In children, this manifests as restlessness, drowsiness, or withdrawal behavior.

### Skin:

The skin often shows redness, wheals (hives) or tissue swelling, especially of the lips or eyes (so-called “angioedema”), even on skin areas that have not had direct contact with the trigger (e.g., after insect bite or consumption of food).

### Gastrointestinal tract:

Nausea, cramping, abdominal pain, vomiting or diarrhea may occur here, as well as an involuntary urge to urinate or defecate.

### Respiratory tract:

Typical signs in the upper respiratory tract are, in addition to runny nose, swelling of the uvula and tongue, as well as tingling and burning in the throat or on the palate, which can sometimes manifest as slurred speech, difficulty swallowing, or wheezing sounds when inhaling. In the lungs, there may be constriction of the bronchi with coughing, shortness of breath, and with whistling sounds (called “wheezing”) and even respiratory failure.

### Cardiovascular system:

Due to a dilation and increased permeability of the vessels, a lot of fluid from the bloodstream can seep into the tissue within a short time, which can lead to dizziness, clouding of consciousness, drop in blood pressure, increase in heart rate (pulse), unconsciousness and even collapse or shock. Direct symptoms to the heart such as arrhythmias are also possible.

## What is the course of anaphylactic reactions?

An anaphylactic reaction can occur within minutes and symptoms can intensify very quickly. However, there are also anaphylactic reactions that build up over the period of an hour and progress slowly. However, a reaction may also spontaneously stop and recede.

The risk and course of recurrent reactions in the future cannot be predicted with certainty. Symptoms may vary in nature, expression, and severity with each reaction.

The severity of a reaction depends on how much and via which route the allergen was ingested, but can also be influenced by nonspecific factors, for example, simultaneous physical stress, psychological stress, acute infections, alcohol or certain medications (so-called augmentation or summation factors).

In addition, underlying diseases such as severe cardiovascular disease, existing and poorly treated asthma, mastocytosis (proliferation of mast cells in various organs of the body), and advanced age can be risk factors for severe reactions. Medications that increase the risk of anaphylaxis include beta-blockers (often prescribed for high blood pressure or cardiac arrhythmias), ACE inhibitors (for high blood pressure), or certain painkillers and rheumatism medications (nonsteroidal anti-inflammatory drugs, NSAIDs).

## What are common triggers of an anaphylactic reaction?

In adults, the most common triggers of anaphylaxis are insect venoms (e.g., wasp or bee), drugs (e.g., painkillers, antibiotics), and foods (e.g., peanuts, hazelnuts, wheat, spices, or shrimp); in children, foods, for example, peanuts, hazelnuts, cashew, cow’s milk, or egg top the list.

## How is anaphylaxis treated?

Treatment is based on whether the reaction is occurring for the first time or the risk of anaphylaxis is known and an emergency kit is already available.

If an emergency kit is available, the procedure is decided according to the organs affected and the severity of the reaction. Roughly, we distinguish between an incipient anaphylaxis (in the passport “yellow”) and a severe reaction (in the passport “red”).

## What medications are available for immediate relief?

Usually, three or four different medications are prescribed individually for the emergency kit for immediate relief, which should be carried together with the anaphylaxis passport as an emergency kit (Table 1).

**Table 1:** Medications for the emergency kit for immediate aid

Drug	Function	Has an effect	Dosage form
Adrenaline	Stabilizes the circulation, expands the respiratory tract, relaxes the intestinal muscles	Few minutes	Auto injector
Antihistamine	Has a decongestant and antipruritic effect. Stabilizes fluid loss into the skin.	About 30 min (depending on preparation and form of preparation)	Liquid, droplets, (melting-/lozenges) tablets
Cortisone	Has an anti-inflammatory, decongestant effect	About 60 min	Juice, suppositories, tablets
Asthma emergency spray (e.g., salbutamol, fenoterol)	Expands the airways (prescribed in case of asthma/known respiratory symptoms)	5–10 min	Spray, powder for inhalation

## How do I recognize a severe anaphylactic reaction?

A severe reaction is defined by the fact that the lungs or cardiovascular system are involved (e.g., in case of respiratory distress or unconsciousness) or two organ systems are affected (e.g., skin with hives and gastrointestinal tract with vomiting). Additional symptoms can be found in the Anaphylaxis Passport and Anaphylaxis Emergency Plan.

## What to do in case of severe reaction and emergency kit available?

1. *Adrenaline: *In the event of a severe reaction, it is most important that the adrenaline auto-injector is used immediately by the affected person or by bystanders or accompanying persons. The epinephrine auto-injector automatically administers a predosed amount of epinephrine into the outer thigh via pressure.
2. *Positioning: *positioning should be performed according to symptoms, that is,
  - for circulatory problems: lie down and slightly raise your legs,
  - in case of unconsciousness: stable lateral position,
  - In case of predominant respiratory symptoms: Upper body upright.
3. *Asthma emergency spray *(if available): If shortness of breath occurs, the bronchodilator asthma emergency spray (e.g., salbutamol, fenoterol) should be given, if available.
4. *Emergency call (112): *According to this, the emergency number 112 should be called immediately to get professional help.
  If two or more people are on the scene, the administration of emergency medication and the placing of the emergency call should be done in parallel.
5. *Antihistamine and cortisone: *After these emergency measures have been taken, the antihistamine (anti-allergic) and cortisone should also be administered.

Further care will then be provided by emergency medical services, with, for example, intravenous access for volume infusion as well as oxygen supply.

In case of severe reactions, inpatient observation for 24 h is recommended, as sometimes symptoms may reappear after 6–8 h (so-called “biphasic reaction”).

## What to do if a mild reaction starts and emergency kit is available?

If there are “only” allergic reactions on the skin, such as hives, redness, wheals, or in the gastrointestinal tract, such as nausea, vomiting, “only” the emergency call can be made at first and the antihistamine and cortisone given, especially if it is unclear whether an allergen ingestion has really occurred. However, if the symptoms worsen, the epinephrine auto-injector should be applied immediately and proceed as described above (severe reaction).

## What to do in case of first anaphylactic reaction when an emergency kit for immediate help is not yet available?

If emergency medication is not yet available for self-help, the emergency call must be made immediately when an anaphylactic reaction is suspected so that treatment can be given as quickly as possible by the medical personnel called in. Patients should be positioned as described above.

## What can be done prophylactically?

After successful treatment of anaphylaxis, it is important to educate those affected about the clinical picture and to strive for identification of the trigger through allergy diagnostics (if possible, referral to an allergist). In addition, the above-mentioned “emergency kit for immediate relief” should be prescribed; the use of the individual medications must be explained in detail and practiced. Recommendations to prevent recurrence of reactions are also important, especially in the case of food allergy, an individually adapted therapeutic elimination diet. Here, advice should be given by a specialized nutritionist. In insect venom allergy, causal treatment by allergen immunotherapy (AIT) should be sought, if possible. In the case of drug allergy, it is important not only to avoid the triggering drugs, but also to test alternative drugs, which is usually done by a provocation test under in-patient conditions.

In addition to the self-help emergency kit, a written plan “Anaphylaxis Passport/Anaphylaxis Emergency Plan” is given for the management of possible new reactions. In this, the prescribed medication is entered by the physician in the weight-adapted dose.

## When is a second epinephrine auto-injector prescribed?

Prescription of a second additional epinephrine auto-injector is recommended for patients with:

- Particularly severe anaphylaxis in the past
- High body weight (over 100 kg)
- Uncontrolled bronchial asthma
- Poor accessibility to the nearest emergency care provider
- Particularly high risk of severe anaphylaxis (e.g. mastocytosis) or for
- Organizational reasons, for example, childcare center, school or according to the family situation.

## Important: the medications for immediate relief must always be with the patient!

At a minimum, the epinephrine auto-injector should either be carried by the person at risk for anaphylaxis or be accessible to a caregiver at all times.

## What else should be considered regarding epinephrine auto-injectors?

The epinephrine auto-injectors currently available in Germany do not differ in substance (epinephrine), but do differ in dose, shelf life, needle length and handling. For this reason, preparations that a patient has been trained to handle should not be interchanged if possible (e.g., for economic reasons by the pharmacist); patients should pay attention to this when the medication is prescribed and, if necessary, the physician be reminded that it is essential to tick the “aut idem” box on the prescription.

For emergency medications, the expiration date must be observed and replacements must be provided in good time. Important for the practice: discard expired products only when the new preparation is available and carried. However, there is a possibility of weaker effect due to expired products.

## Where can I get more information, training, contacts?

### AGATE anaphylaxis training:

Standardized and quality-controlled training programs are offered through “Arbeitsgemeinschaft Anaphylaxie—Training und Edukation” (AGATE). The training focuses on the practical aspects of self-treatment, with role-plays including use of the epinephrine auto-injector, allergen avoidance strategies and coping with fear of re-allergen contact. Age-appropriate training is given to the patients themselves, but also to their parents and caregivers.

### German Allergy and Asthma Association (DAAB):

As a patient organization, the DAAB is the contact for questions about everyday management with anaphylaxis (allergen avoidance, daycare, school, travel, etc.) In addition to the standardized anaphylaxis passport/emergency plan (see below), other information materials (brochures, shopping guides, restaurant cards, certificates for air travel, etc.) and aids (see below) can be obtained from the DAAB. Online seminars on everyday and emergency management of anaphylaxis are offered for patients, parents as well as daycare centers and schools, and contacts such as allergy-specialized nutritionists can be found.

## Resources

### Auto-injector trainer (training pen):

This is an adrenaline auto-injector without medication and needle, with the help of which the application and handling can be practiced. They are available from all manufacturers of epinephrine auto-injectors and can be obtained free of charge from them as well as from pharmacies or the DAAB.

### Anaphylaxis passport:

Document issued by a doctor containing information on the person concerned, the prescribed medication, and on the recognition of anaphylactic reactions and the measures to be taken in an emergency. It is kept together with the medication and always carried along.

### Anaphylaxis emergency plan:

Document with the same information as the anaphylaxis passport in DIN A4 format to pass on to third parties (e.g., daycare center, school, employer) or the other environment.

### Storage bags:

They are available through some epinephrine auto-injector manufacturers, the DAAB or via the Internet.

## Addendum: a few words about Covid-19 vaccination and allergy

Anaphylactic reactions after vaccination are possible but very rare, for COVID-19 vaccines at a frequency of 1–10 per 1 million.

In case of known anaphylaxis against vaccines, drugs—especially injected substances—or if the trigger is unclear, prior allergological clarification is recommended.

Do not vaccinate individuals with anaphylaxis to ingredients of the intended vaccine, for example, polyethylene glycol or tromethamine.

For most allergy sufferers, COVID-19 vaccination is safe.

## Abbreviations

ACE: Angiotensin-converting enzyme inhibitors (drugs for high blood pressure and heart failure)
AGATE: Arbeitsgemeinschaft Anaphylaxie – Training und Edukation (“working group anaphylaxis—training and education”)
AIT: Allergen immunotherapy
AMWF: Association of Scientific Medical Societies
COVID-19: Coronavirus disease 2019
DAAB: German Allergy and Asthma Association (patient organization)
DGAKI: German Society for Allergology and Clinical Immunology
NSAIDs: Nonsteroidal anti-inflammatory drugs
